# Draft Genome Sequence of *Bacillus litoralis* C44, Isolated from Chinese Scholar Tree (*Sophora japonica*) Forest Soil

**DOI:** 10.1128/genomeA.01059-16

**Published:** 2016-09-29

**Authors:** Weili Lyu, Yunlong Wu, Ya Liu, Zhitang Lyu

**Affiliations:** a College of Life Sciences, Hebei University, Baoding, People’s Republic of China; b Key Laboratory of Microbial Diversity Research and Application of Hebei Province, Baoding, People’s Republic of China; c Key Laboratory of Medicinal Chemistry and Molecular Diagnosis (Hebei University), Ministry of Education, Baoding, People’s Republic of China

## Abstract

*Bacillus litoralis* C44 can hydrolyze rutin to produce isoquercetin by the enzyme α-l-rhamnosidase. We report here the genome sequence and annotation result of strain C44. The genomic information will serve as references to the physiology, genetics, and evolution of this species and further genetic engineering research in this species.

## GENOME ANNOUNCEMENT

*Bacillus litoralis* is a Gram-positive, motile, endospore-forming, rod-shaped bacterium. This species was first isolated and described by Jung-Hoon Yoon in 2005 (1). However, there are no more reports about whole-genome information of this species until today. *Bacillus litoralis* C44 was isolated by Yating Zhang in 2010 (38°52′0″N 115°30′00″ E). This strain can hydrolyze rutin to isoquercetin efficiently and specifically by coding α-l-rhamnosidase (2). To find the genes of α-l-rhamnosidase and probe the mechanism of enzyme specificity, a whole-genome shotgun sequencing strategy was carried out.

The whole-genome DNA was extracted from bacterial overnight cultures by a phenol-chloroform method (3) and then was sequenced by a 2 × 100-bp paired-end approach using the Illumina HiSeq TM2000 platform at Novogene (Tianjin, China). *De novo* genome assembly was performed using SOAPdenovo2 version 2.04 (4), which generated 46 contigs with an *N_50_* value of 248,449 bp and a largest contig size of 771,368 bp. Then, gene prediction was performed with GeneMarkS version 3.25 (5), while tRNA was predicted with tRNAscan-SE version 1.3.1 (6), rRNA was predicted with RNAmmer version 1.2 (7), small RNA (sRNA) was predicted with Rfam version 12.0 (8), and short tandem repeats (STR) were predicted with Repeat Masker version 4.05. Finally, the gene functions were annotated into the COG (9), KEGG (10), NR databases with BLASTx, respectively, and the GO database with InterProScan 5 (11).

The final assembled genome is 5,616,296 bp and has a G+C content of 35.54%. The organism possesses 5,403 coding sequences (CDSs), which yielded a coding capacity of 4,696,614 nucleotides, 115 predicted tRNA, nine predicted sRNA, and 208 predicted STR. The coding density of the draft genome is 83.62%, with an average gene length of 869 bp. According to the annotation of the whole-genome sequence of the four databases mentioned above, there are four α-l-rhamnosidase genes predicted, which were designated ORF0334, ORF0338, ORF2809, and ORF2811, respectively. ORF0334 and ORF0338 are located on contig 1 (accession no. LWSG01000001), while both ORF2809 and ORF2811 are located on contig 8 (accession no. LWSG01000045). Here, we announce the first genome sequence of a *Bacillus litoralis* strain.

### Accession number(s).

This whole-genome shotgun project has been deposited at DDBJ/ENA/GenBank under the accession no. LWSG00000000. The version described in this paper is version LWSG01000000.
